# Strengthening Uganda’s Climate-Resilient Health Systems: A Position Paper on Climate and Health Policy

**DOI:** 10.3389/phrs.2026.1609302

**Published:** 2026-04-23

**Authors:** Bernard Jackson Zikanga

**Affiliations:** Seed Global Health, Boston, UG, United States

**Keywords:** climate -resilient health systems, health systems strengthening, public health diplomacy, domestic health financing, and climate change adaptation

## Introduction

Climate change is already impacting health outcomes in Uganda. Rising temperatures, erratic rainfall, floods, and droughts have increased the incidence of climate-sensitive diseases such as malaria, cholera, and respiratory infections [1]. Nearly half of Uganda’s health facilities are located in areas prone to floods or droughts, with over 70% experiencing interruptions in service delivery during climate-related disasters [2]. The combined effects of environmental degradation, food insecurity, and forced migration compound the burden on an already fragile health system. These challenges underscore the urgent need for integrating climate adaptation into health sector policies, workforce development, and financing structures.

Uganda’s approach to integrating climate change into health policy also reflects a growing dimension of public health diplomacy aligning national adaptation priorities with global climate and health commitments through negotiation, policy alignment, and cross-sector partnerships.

## Discussion

Uganda’s policy landscape provides a strong foundation for climate–health integration. The Health National Adaptation Plan (HNAP 2025–2030) outlines strategies for building resilience in health service delivery, workforce training, infrastructure, and surveillance [3]. The Nationally Determined Contribution (NDC3.0), expected in 2025, offers an opportunity to embed measurable health adaptation indicators within national climate commitments [4].

These multisectoral efforts illustrate health diplomacy in practice, where technical adaptation actions intersect with governance, partnership-building, and dialogue between domestic institutions and international frameworks such as the United Nations Framework Convention on Climate Change (UNFCCC) and WHO’s climate–health agenda [5].

Health workforce capacity remains a cornerstone of climate adaptation. Training health professionals to anticipate, prevent, and manage climate-related diseases improves the system’s responsiveness. Integrating climate–health modules into pre-service and in-service curricula is a practical first step [6]. Similarly, climate-sensitive budgeting ensures predictable financing for adaptation. Evidence shows that *ad hoc* or project-based funding leads to inefficiencies and poor sustainability [7]. Domestic financing mechanisms and tagged budget lines enable better tracking of resources, accountability, and integration with broader health financing reforms.

Strengthening data and surveillance systems is equally essential. Uganda’s District Health Management Information System version two (DHIS2) offers a strong platform for integrating climate and environmental indicators. Pilot initiatives such as the DHIS2 Climate App demonstrate feasibility for combining climate and health data to enhance early warning and response [8]. However, scaling such innovations nationally requires standardized indicators, multisectoral data sharing, and sustained technical capacity. Robust monitoring, reporting, and verification (MRV) systems would improve national planning and international reporting under the UNFCCC [9].

## Recommendations

1. Integrate climate–health adaptation into health workforce training and continuous professional development programs.
2. Establish dedicated budget lines for climate adaptation within the Ministry of Health and district plans to ensure sustained financing.
3. Strengthen surveillance and monitoring systems to include climate and environmental indicators.
4. Foster cross-sectoral collaboration between health, environment, water, agriculture, and disaster risk management agencies. This collaboration reflects practical health diplomacy, where local and international stakeholders work together to strengthen resilience.
5. Enhance community engagement to build awareness and local resilience through participatory planning and risk communication.

### Conclusion

Uganda’s transition toward a climate-resilient health system depends on bridging the gap between policy commitments and implementation. Operationalizing HNAP and integrating health into the NDC 3.0 process present unique opportunities to align national adaptation goals with health system strengthening [10]. Building resilience in the health sector is both a climate and diplomatic imperative, linking Uganda’s domestic health priorities with international frameworks and cooperation under global climate governance.

## References

[1] Ministry of Health. Climate Vulnerability and Adaptation Assessment for the Health Sector. Kampala: MoH (2023).

[2] Uganda National Meteorological Authority. Climate Trends and Projections Report. Kampala: UNMA (2023).

[3] Ministry of Health. Health National Adaptation Plan (HNAP 2025–2030). Kampala: MoH (2025).

[4] Ministry of Water and Environment. Uganda’s Nationally Determined Contribution (NDC 3.0) Draft. Kampala: MWE (2025).

[5] World Health Organization. Operational Framework for Building Climate-Resilient Health Systems. Geneva: WHO (2021).

[6] Government of Uganda. Vision 2040. Kampala: National Planning Authority (2013).

[7] World Bank. Climate Change and Health Diagnostics in Sub-Saharan Africa. Washington DC: World Bank (2022).

[8] Uganda Bureau of Statistics. Statistical Abstract 2023. Kampala: UBOS (2023).

[9] Rockefeller Foundation. Building Climate-Resilient Health Systems in Africa. New York: Rockefeller Foundation (2022).

[10] Ministry of Health. Health Sector Development Plan (HSDP III 2020/21–2024/25). Kampala: MoH (2020).

